# The ABC (Age, Bystander, and Cardiogram) score for predicting neurological outcomes of cardiac arrests without pre-hospital return of spontaneous circulation: A nationwide population-based study

**DOI:** 10.1016/j.resplu.2024.100673

**Published:** 2024-05-31

**Authors:** Kazuyuki Uehara, Takashi Tagami, Hideya Hyodo, Gen Takagi, Toshihiko Ohara, Masahiro Yasutake

**Affiliations:** a Department of General Medicine and Health Science, Nippon Medical School, Tokyo, Japan; b Department of Emergency and Critical Care Medicine, Nippon Medical School Musashi-Kosugi Hospital, Kanagawa, Japan

**Keywords:** ABC score, Out-of-hospital cardiac arrest, Neurological outcome, Termination of resuscitation, Extracorporeal cardiopulmonary resuscitation

## Abstract

**Aim:**

We previously proposed the ABC score to predict the neurological outcomes of cardiac arrest without prehospital return of spontaneous circulation (ROSC). Using nationwide population-based data, this study aimed to validate the ABC score through various resuscitation guideline periods.

**Methods:**

We analysed cases with cardiac arrest due to internal causes and failure to achieve prehospital ROSC in the All-Japan Utstein Registry. Patients from the 2007–2009, 2012–2014, and 2017–2019 periods were classified into the 2005, 2010, and 2015 guideline groups, respectively. Neurological outcomes were assessed using cerebral performance categories (CPCs) one month after the cardiac arrest. We defined CPC 1–2 as a favourable outcome. We evaluated the test characteristics of the ABC score, which could range from 0 to 3.

**Results:**

Among the 162,710, 186,228, and 190,794 patients in the 2005, 2010, and 2015 guideline groups, 0.7%, 0.8%, and 0.9% of the patients had CPC 1–2, respectively. The proportions of CPC 1–2 were 2.9%, 3.6%, and 4.6% in patients with ABC scores of 2 and were 9.5%, 13.3%, and 16.8% in patients with ABC scores of 3, respectively. Among patients with ABC scores of 0, 0.2%, 0.1%, and 0.2%, all had CPC 1–2, respectively. The areas under the receiver operating characteristic curves for the ABC score were 0.798, 0.822, and 0.828, respectively.

**Conclusions:**

The ABC score had acceptable discrimination for neurological outcomes in patients without prehospital ROSC in the three guideline periods.

## Introduction

The neurological prognosis for out-of-hospital cardiac arrest (OHCA) remains poor.^1^ Approximately 90% of the patients with OHCA reach hospitals without return of spontaneous circulation (ROSC).^2^ In these patients, the recovery rate with a favourable neurological status is still less than 1%.^3^

If ROSC is not achieved in the prehospital setting, the clinician must make decisions such as terminating resuscitation or introducing extracorporeal cardiopulmonary resuscitation (ECPR) with limited time and information.^4^ The termination of resuscitation requires scientific identification of patients with poor prognoses.^5^ ECPR has been reported to improve prognosis;^6^, ^7^ however, this treatment is resource-intensive and requires patient selection.^5^, ^8^ To make these clinical decisions, prognostic evaluation at hospital arrival is crucial.^4^, ^9^

**Fig. 1:**
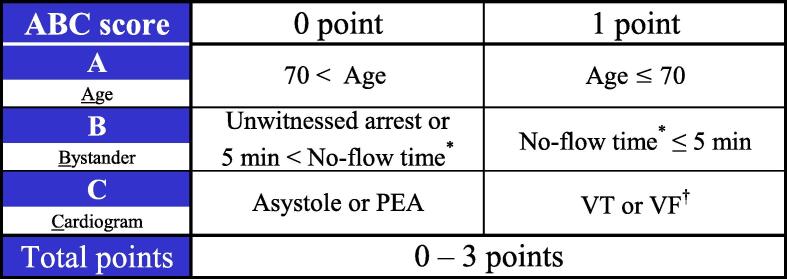
**The ABC score.** This figure was reproduced from [Uehara, K, Tagami, T, Hyodo, H, et al. Prehospital ABC (Age, Bystander, and Cardiogram) scoring system to predict neurological outcomes of cardiopulmonary arrest on arrival: post hoc analysis of a multicentre prospective observational study. Emergency Medicine Journal 2023;40:42–47] with permission from BMJ Publishing Group Ltd. *No-flow time was defined as the interval from witnessed arrest to first chest compression. ^†^VT or VF indicates that initially monitored rhythm as those or performed public access defibrillation. VF, ventricular fibrillation; VT, ventricular tachycardia; PEA, pulseless electrical activity.

Therefore, there is an urge for a simple, quick, and reliable prognostic assessment tool for patients without prehospital ROSC.^4^ For this purpose, we developed an ABC scoring system (Fig. 1).^10^ The ABC score consists of three variables: age (≤70 years), bystander factor (no-flow time ≤5 min), and cardiogram (ventricular tachycardia or fibrillation as initial rhythm). As these variables are essential for resuscitation, the ABC score is considered usable worldwide. However, this score was validated only in a limited region and period, which limits its generalizability.

Before the clinical use of the ABC score, it should be evaluated in other populations under different time periods.^11^, ^12^ This study aimed to validate the ABC score through different resuscitation guideline periods using nationwide population-based data.

## Methods

### Study design and population

We evaluated the ABC score using the All-Japan Utstein Registry.^13^ This registry is a nationwide, population-based, prospective observational study enrolling all patients involved in emergency medical services for OHCA in Japan. The Fire and Disaster Management Agency launched a registry in 2005 and recorded the OHCA data according to the Utstein style.^14^ As of 2024, patient enrolment will continue, and data from 2005 to 2021 will be available for analysis. During this period, international resuscitation guidelines were revised in 2005, 2010, 2015, and 2020.^5^, ^15^ We divided patients into guideline groups to validate the ABC score throughout the three different guideline periods. The revision and subsequent years were considered transition periods and excluded from the analysis. Therefore, we analysed data from patients with OHCA during 2007–2009, 2012–2014, and 2017–2019 in the registry.

This study was approved by the institutional review board of Nippon Medical School Hospital (No. B-2020-207). No informed consent was required as the patient data were de-identified.

### Selection of participants

The All-Japan Utstein Registry covers all areas of Japan with a population of approximately 127 million. On the other hand, the SOS-KANTO 2012,^16^ which was used to derive the ABC score, covers one region of Japan, the Kanto area, with a population of 40 million. To validate the score externally, we used a population that was completely different from the population in which the score was developed. Therefore, we excluded patients in the region covered by SOS-KANTO 2012.

We enrolled patients who were 18 years of age and had been subjected to resuscitation attempts. Patients were excluded if they had cardiac arrest due to external causes (such as trauma, accidental hypothermia, drowning, and poisoning), if their ABC score could not be calculated due to missing values, if their neurological outcomes was unknown, or if they achieved prehospital ROSC. Patient data from 2007 to 2009, 2012 to 2014, and 2017 to 2019 were categorized into the 2005, 2010, and 2015 guidelines groups, respectively.

### Emergency medical system in Japan

In Japan, ambulance crews can perform cardiopulmonary resuscitation and defibrillation. In addition to these resuscitation procedures, well-trained paramedics are allowed to intubate the endotracheal tube and administer epinephrine in a prehospital setting. Emergency medical services personnel are prohibited to terminate resuscitation unless the patient is obviously dead.^17^ Therefore, the ABC score variable does not affect the decision to terminate prehospital resuscitation and to cancel hospital transport.

### Measurement and outcomes

The ABC score consists of three variables: age, bystander factor, and cardiogram(electrocardiogram). If the patient is 70 years or younger, he or she receives one point for factor A. Regarding the B factor, a patient is assigned one point if their no-flow time is 5 min or less. The no-flow time is defined as the interval from the moment of collapse to the first chest compression, which is only available for witnessed arrest. A patient whose arrest was not witnessed is assigned zero points for factor B. Regarding the C factor, if the first monitored rhythm is ventricular tachycardia or fibrillation, the patient gets one point. A patient who has received public access defibrillation is also assigned one point for the C factor. The maximum ABC score is 3, and the minimum is 0. The factors comprising the ABC score of each case (age, witness, no-flow time, and initial cardiac rhythm) were determined by the dispatched ambulance crews.

Neurological outcomes were assessed by the physician at each hospital one month after cardiac arrest using cerebral performance categories (CPCs).^18^ The CPC ranges from 1 to 5 (1: good cerebral performance or mild cerebral disability; 2: moderate cerebral disability; 3: severe cerebral disability; 4: coma or vegetative state; and 5: death). We considered CPC 1 and 2 to be favourable neurological outcomes.^19^

### Analysis

We evaluated the sensitivity, specificity, and positive/negative likelihood ratio using the ABC score. The discrimination of the scores was also confirmed using the areas under the receiver operating characteristic curves for each guideline group. The optimum cut-off value of the score was determined using the Youden index (sensitivity + specificity − 1).^20^ The calibration was confirmed graphically by plotting the predicted and observed probabilities. All analyses were performed using the IBM SPSS Statistics version 27 (Armonk, NY, USA).

## Results

### Study population

**Fig. 2:**
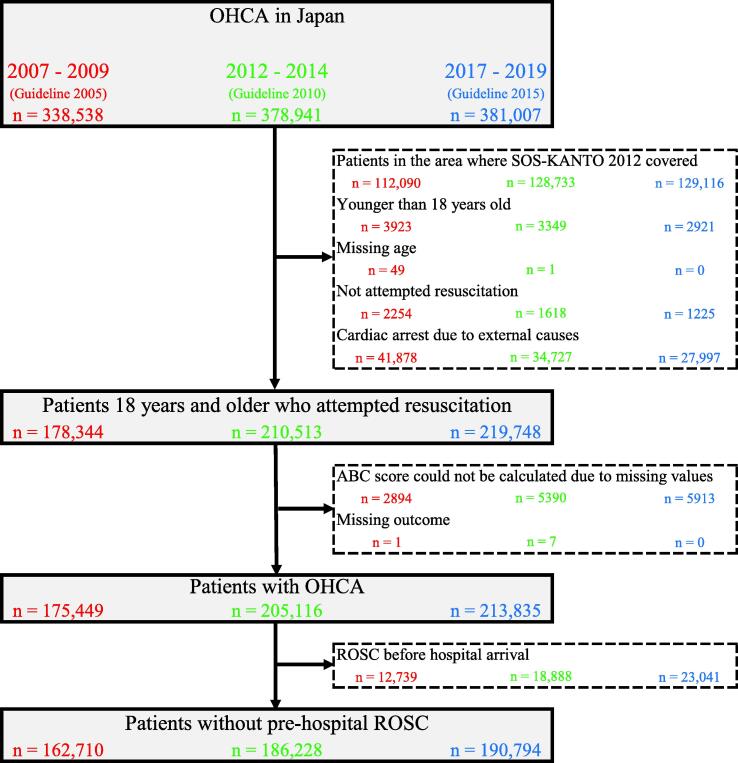
**Flow chart of the patient selection.** OHCA, out-of-hospital cardiac arrest; ROSC, return of spontaneous circulation; SOS-KANTO 2012, Survey of survivors after out-of-hospital cardiac arrest in the Kanto area in 2012.

During 2007–2009, 2012–2014, and 2017–2019, 338,538, 378,941, and 381,007 OHCA patients were enrolled in the All-Japan Utstein Registry, respectively. Upon applying the inclusion and exclusion criteria, 162,710, 186,228, and 190,794 patients without prehospital ROSC were selected for analysis and divided into the 2005, 2010, and 2015 guideline groups, respectively (Fig. 2).

Table 1 shows the patient characteristics and numbers assigned to each ABC score based on the three guideline groups. The proportions of CPC 1 and 2 in the 2005, 2010, and 2015 guideline groups were 0.7%, 0.8%, and 0.9%, respectively.

**Table 1:** Patients characteristics in Guideline groups 2005, 2010, and 2015 without prehospital ROSC.

	Guideline 2005 group	Guideline 2010 group	Guideline 2015 group
	*n* = 162,710	*n* = 186,228	*n* = 190,794
Year of cardiac arrest	2007–2009	2012–2014	2017–2019
Age, years	79 (68–86)	81 (71–87)	81 (71–88)
Sex, male	92,868 (57.1)	102,688 (55.1)	105,468 (55.3)
Witnessed cardiac arrest	62,842 (38.6)	69,834 (37.5)	71,456 (37.5)
Bystander CPR	74,040 (45.5)	93,833 (50.4)	105,954 (55.5)
No-flow time^a^, min	4 (0–10)	3 (0–10)	3 (0–10)
Interval from call dispatch to patient contact, min	8 (6–10)	9 (7–11)	9 (7–11)
Interval from patient contact to hospital arrival, min	20 (16–26)	21 (16–28)	21 (16–28)
First monitored rhythm by emergency medical service^b^			
VT or VF	11,636 (7.2)	10,729 (5.8)	9830 (5.2)
PEA or asystole	146,182 (89.8)	171,295 (92.0)	177,758 (93.2)
Public access defibrillation	848 (0.5)	1764 (0.9)	2083 (1.1)
ABC score variables			
A: Age ≤ 70 years	46,695 (28.7)	46,309 (24.9)	45,274 (23.7)
B: No-flow time^a^ ≤ 5 min	35,589 (21.9)	40,800 (21.9)	42,737 (22.4)
C: VT or VF as initial rhythm^c^	12,290 (7.6)	12,113 (6.5)	11,475 (6.0)
ABC scores			
0 points	87,474 (53.8)	105,611 (56.7)	109,650 (57.5)
1 point	58,603 (36.0)	64,327 (34.5)	65,166 (34.2)
2 points	13,928 (8.6)	13,975 (7.5)	13,614 (7.1)
3 points	2705 (1.7)	2315 (1.2)	2364 (1.2)
Neurological outcomes			
CPC 1–2	1161 (0.7)	1420 (0.8)	1801 (0.9)
CPC 3–4	2558 (1.6)	2630 (1.4)	2563 (1.3)
CPC 5	158,991 (97.7)	182,178 (97.8)	186,430 (97.7)

Categorical variables are presented as numbers (percentages). Continuous variables are presented as median (interquartile range).

A, Age; B, Bystander factor; C, Cardiogram; CPC, cerebral performance category; CPR, cardiopulmonary resuscitation; PEA, pulseless electrical activity; ROSC, return of spontaneous circulation; VF, ventricular fibrillation; VT, ventricular tachycardia.

a No-flow time is defined as the interval from the moment of collapse to the first chest compression (obtainable only for a witnessed arrest).

b Total percentages of the first monitored rhythm were not equal to 100% because of missing values and rhythm with spontaneous circulation.

c VT or VF indicates that initially monitored rhythm as those or performed public access defibrillation.

### Discrimination and calibration of the ABC score

**Fig. 3:**
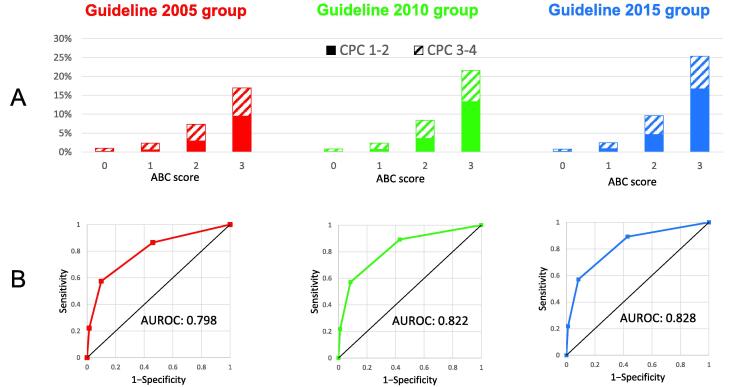
**The ABC scores in the guideline groups 2005, 2010, and 2015.** The guideline groups 2005, 2010, and 2015 are represented in red, green, and blue, respectively. (A) Proportions of patients with CPC 1**–**2 and 3**–**4 according to ABC score. Filled areas represent CPC 1**–**2 and shaded areas represent CPC 3**–**4. (B) The receiver operating characteristic curves of the ABC score. AUROC, area under the receiver operating characteristic curve; CPC, cerebral performance category.

In the guideline groups of 2005, 2010, and 2015, the proportions of CPC1-2 among patients with an ABC score of 2 were 2.9%, 3.6%, and 4.6% respectively. The proportions of CPC1-2 among patients with the score of 3 were 9.5%, 13.3%, and 16.8%, respectively (Fig. 3A). In contrast, among patients with the score of 0, the proportions of CPC 1–2 were 0.2%, 0.1%, and 0.2%, whereas those of CPC 3–4 were 0.8%, 0.7%, and 0.6% in the guideline groups of 2005, 2010, and 2015, respectively.

Table 2 lists the test characteristics of the ABC scores. According to the 2005, 2010, and 2015 guidelines, the positive likelihood ratios for 2 or more points were 5.80, 6.82, and 7.22, respectively, and the positive likelihood ratios for 3 points were 14.67, 20.05, and 21.12, respectively. The ABC score areas under the receiver operating characteristic curves were 0.798, 0.822, and 0.828 in the 2005, 2010, and 2015 guideline groups, respectively (Fig. 3B). The optimal cut-off score determined by the Youden index was consistently two points.

**Table 2:** Discrimination of the ABC score for CPC 1–2 in the guideline groups 2005, 2010, and 2015 without prehospital ROSC.

	Guideline 2005 group			Guideline 2010 group			Guideline 2015 group		
ABC score	1≤	2≤	3	1≤	2≤	3	1≤	2≤	3
*n*	75,236	16,633	2705	80,617	16,290	2315	81,144	15,978	2364
CPC 1–2, *n*	1004	666	258	1266	811	309	1614	1029	396
CPC 1–2, %	1.33 (1.25–1.42)	4.00 (3.71–4.31)	9.54 (8.46–10.71)	1.57 (1.49–1.66)	4.98 (4.65–5.32)	13.35 (11.99–14.80)	1.99 (1.89–2.09)	6.44 (6.06–6.83)	16.75 (15.27–18.32)
Sensitivity, %	86.48 (84.37–88.39)	57.36 (54.46–60.23)	22.22 (19.86– 24.73)	89.15 (87.42–90.73)	57.11 (54.49–59.71)	21.76 (19.64–24.00)	89.62 (88.12–90.99)	57.13 (54.81–59.44)	21.99 (20.09–23.97)
Specificity, %	54.05 (53.81–54.29)	90.12 (89.97–90.62)	98.49 (98.42–98.54)	57.06 (56.84–57.29)	91.62 (91.50–91.75)	98.91 (98.87–98.96)	57.92 (57.70–58.14)	92.09 (91.97–92.21)	98.96 (98.91–99.00)
Positive likelihood ratio	1.88 (1.84–1.93)	5.80 (5.51–6.11)	14.67 (13.08–16.45)	2.08 (2.04–2.12)	6.82 (6.50–7.15)	20.05 (18.00–22.33)	2.13 (2.09–2.17)	7.22 (6.92–7.54)	21.12 (19.15–23.28)
Negative likelihood ratio	0.25 (0.22–0.29)	0.47 (0.44–0.51)	0.79 (0.77–0.81)	0.19 (0.16–0.22)	0.47 (0.44–0.50)	0.79 (0.77–0.81)	0.18 (0.16–0.21)	0.47 (0.44–0.49)	0.79 (0.77–0.81)

Numbers in parentheses indicate 95% confidence intervals.

CPC, cerebral performance category; ROSC, return of spontaneous circulation.

**Fig. 4:**
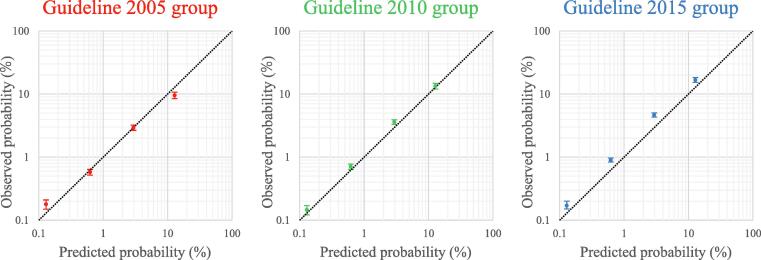
**Calibration plots of the ABC scores in the guideline groups 2005, 2010, and 2015.** Dotted lines indicate perfect calibration, and the I bars show 95% confidence intervals of the observed probabilities.

The calibration plots revealed that the predicted probabilities closely matched the observed probabilities at all points of the ABC score in the three guideline groups (Fig. 4).

## Discussion

In three existing guideline periods, the areas under the receiver operating characteristic curves of the ABC score were consistently around 0.80 (Fig. 3B), indicating acceptable discrimination. The calibration of the score was good at all time points. These results suggest that the ABC score had good predictability for neurological outcomes of patients without prehospital ROSC in the Japanese nationwide population-based data.

The positive likelihood ratio for CPC 1–2 with an ABC score >2, which was consistently the optimal cut-off, exceeded 5 (Table 2). Moreover, the positive likelihood ratio for CPC 1–2 with an ABC score of 3 was >10, which is generally considered a good clinical prediction test for CPC 1–2.^21^ These results indicate that patients with potentially favourable neurological outcomes can be identified among those without prehospital ROSC using the ABC score, especially in the case of 3 points.

In contrast, patients with an ABC score of 0 had less than a 0.2% chance of having a CPC of 1–2 in these study periods (Fig. 3A). Additionally, the proportion of patients with CPC 3–4 was about four times that of patients with CPC 1–2. These results suggest that the neurologic outcome is likely to be poor even if spontaneous circulation is restored at the hospital in patients with an ABC score of 0.

The percentage of cases with bystander cardiopulmonary resuscitation increased throughout the study period, which might have improved the neurological outcomes (Table 1).^22^ Despite such variations, the ABC score consistently showed good predictive accuracy over the three guideline periods. To ensure sustained accuracy over time, one of the contributing factors lies in the variables that comprise the score. Age,^4^, ^23^, ^24^, ^25^, ^26^, ^27^ no-flow time,^27^, ^28^, ^29^, ^30^, ^31^, ^32^ and initial cardiac rhythm^4^, ^23^, ^24^, ^27^, ^31^, ^32^, ^33^, ^34^, ^35^ have been identified as predictors of favourable outcomes among OHCA in various populations and eras. Therefore, their combination into a single score may be a reason for the observed consistency performance.

Several clinical prediction rules for outcomes of OHCA have already been proposed. The basic life support termination of resuscitation rule has been implemented at the scene.^36^ However, it cannot be used in countries where prehospital termination of resuscitation is legally prohibited. To terminate resuscitation at hospital arrival, another Japanese study proposed a rule for use in the emergency department.^37^ These termination rules predict death, whereas the ABC score predicts neurological outcomes. The SWAP scores (shockable, witnessed, age, and pH) were also proposed to predict OHCA neurological outcomes without prehospital ROSC.^4^ However, the ABC score has several advantages over the SWAP score. First, the SWAP score has been validated only in a single hospital, whereas the ABC score has been validated in nationwide population-based data. Second, the SWAP score includes blood pH, which limits its application to only after arrival at the hospital. In contrast, the ABC score can be calculated in a prehospital setting because all variables can be obtained at the scene.

Specifically, the ABC score might have some merits in clinical situations. First, the ABC score may be useful in triaging patients for ECPR. This score allows for on-scene selection of candidates for ECPR, whose cardiac arrests are potentially reversible and more likely to have favourable neurological outcomes despite not achieving prehospital ROSC^5^. Thus, these patients can be transported to hospitals where ECPR is available. The score also enables emergency physicians to prepare ECPR before hospital arrival and minimize the low-flow time (interval from first chest compression to ROSC), which is a predictor of favourable outcome.^38^, ^39^, ^40^, ^41^, ^42^, ^43^ In addition, the ABC score is composed of patient age,^39^, ^44^ bystander factor,^45^, ^46^, ^47^ and cardiogram,^47^ all of which are predictors of favourable outcomes in ECPR. Furthermore, a previous study showed that patients who underwent ECPR with higher ABC scores had good outcomes.^10^ Therefore, the score can be expected to play a potential role in selecting patients for ECPR and improving their prognosis. Second, in contrast, an ABC score of 0 is a sign of poor prognosis and is useful when considering termination of resuscitation at the hospital. This simple, quick, and reliable ABC score might enable clinicians to predict neurological outcomes and help them decide on treatment plans during ongoing resuscitation.

This study had some limitations. First, the ABC score does not cover in-hospital cardiac arrest and OHCA, in which spontaneous circulation is restored in a prehospital setting. Second, prehospital care systems for cardiac arrest differ around the world. Therefore, validation is required before implementing the ABC score outside of Japan. Third, due to the nature of retrospective study, the influence of self-fulfilling prophecies cannot be ruled out. Lastly, although the ABC score was useful in identifying the neurological outcome of patients without prehospital ROSC, it is unclear whether the score is appropriate as an indication criterion for ECPR. Hence, further research is required to resolve this issue. Furthermore, even if the ABC score is appropriate as an indication criterion for ECPR, the optimal cut-off point for selecting patients for ECPR is unknown. Setting an appropriate point for ECPR requires additional research to confirm treatment outcomes and cost-effectiveness.

## Conclusions

The ABC score showed acceptable discrimination and calibration in Japanese nationwide population-based OHCA data. High scores, especially 3 points, indicated a high likelihood of favourable outcomes, whereas a score of 0 was a sign of unfavourable outcomes. These results were consistent in the three existing guideline periods.

## CRediT authorship contribution statement

**Kazuyuki Uehara:** Writing – review & editing, Writing – original draft, Visualization, Project administration, Methodology, Investigation, Conceptualization. **Takashi Tagami:** Writing – review & editing, Validation, Supervision, Methodology, Conceptualization. **Hideya Hyodo:** Writing – review & editing, Supervision, Software, Resources. **Gen Takagi:** Writing – review & editing, Supervision, Software, Resources. **Toshihiko Ohara:** Writing – review & editing, Supervision, Software, Resources. **Masahiro Yasutake:** Writing – review & editing, Supervision, Software, Resources.

## Declaration of competing interest

The authors declare that they have no known competing financial interests or personal relationships that could have appeared to influence the work reported in this paper.
